# An Unobjectionable Form for the Administration of Medicines

**Published:** 1884-08

**Authors:** M. S. French


### An Unobjectionable Form for the Administration of Medicines.

Of all the various forms in which medicine is prescribed, there is none so well suited for the administration of drugs possessing a poisonous influence, unpleasant odor, or taste, as the manufactured pill form.

The advantages derived from prescribing remedies in this form are so apparent, that it was a matter of surprise to learn that their use was not more general. It is on this account that I present this short article, hoping that it may lead to more extensive use and appreciation of this valuable method of prescribing.

Manufactured pills will become more popular with physicians as their value and advantages are recognized. They are so beautiful in appearance, so accurately compounded, so quickly dispensed, reducing the danger of making a mistake, on the part of the physician or druggist, to an almost impossibility.

They also enable the physician to carry with him, in a small and compact form, ready for administration, all the remedies that might be needed in emergencies, and night practice.

To the physician engaged in country practice, where his patients are at a distance from a drug store, they become of especial value.

In that class of pharmaceutical preparations which exhibits medicines in an elegant and palatable form, without the sacrifice of quality, uniformity or accuracy, American pharmacists have been doing excellent work, the result being that most of the objectionable features which formerly pertained to manufactured pills have been removed, being now produced in such a perfect and attractive form, that the most fastidious patient can find no objection to urge against their use.

There is little doubt but that much of the popularity of Hahnemann's system of cure, based upon the maxim *similia similibus curantur*, is due, not to the truth of the law, but to the preference for the tiny, tasteless and attractive pellets; and the sooner the medical profession recognizes this, the quicker will the public see that, aside from pleasant doses, there is nothing but fallacy and error in homœopathy.

There are some drugs used in practice that cannot be administered in pill form, and there are instances where solutions are preferable. Such is the case in treating the various types of fevers, and where tonics are employed; but in the greater number of cases, the object desired is a continued influence, rather than an immediate impression upon the system, and for the accomplishment of this end pills are best suited.

In an article of this nature, it is not necessary or possible to dwell upon all the conditions to which the administration of remedies in the manufactured pill form are applicable, but to detail a few of the more frequent diseases, and specify instances where they have been used with success, during the past six or seven years.

The physician engaged in city practice sees no departures from health so frequently, as those associated with the digestion and assimilation of food. A majority of the patients presenting themselves for treatment, suffer from either a simple dyspepsia or some one of its many manifestations. The life led by people massed together in large cities is such an artificial one, the inhabitants being so subjected to the enervating influences surrounding them, that the indiscretions of diet, mode of living, anxieties, and the continual struggle for existence, become active factors in producing the large number of dyspeptic cases.

There are certain conditions that cannot be relieved by dietetic and hygienic treatment alone, but require the use of drugs, which should be given in the most pleasant and unobjectionable form; as there is no class of patients so prone to find fault with their medicines, being best suited by doses that are attractive, tasteless and not bulky.

It will not be a digression to dwell upon the treatment of this common affection, as it will enable me to mention more particularly the remedies suited for administration in the form under consideration.

The power of digestion being depressed, special aids to that function will be found in pepsin or alkalies; the anæmic condition may require iron, quinine or strychnia. Where remedies are needed, the action of which must be sedative, good results may be obtained from bismuth, opium, belladonna, arsenic or silver.

A very useful pill is that of aloin (gr. $\frac{1}{5}$) and strychnia (gr. $\frac{1}{60}$) given at bed-time; this will produce a mild, thorough, laxative effect, unloading the stomach and intestines, removing the fetid breath, the furred coating upon the tongue, and the cerebral congestion.

An error liable to be committed is the administration of cathartics of too active a nature; they are rarely called for, do little good, and might possibly do evil. Mild laxatives only are required, and care should be exercised as to their selection and frequency.

Formerly it was my custom to use pepsin and pancreatine more than at present, as recent investigation has led me to believe that as much benefit can be derived from the former agent alone, as when given in combination with the latter. Pancreatine has been shown to be itself digested by pepsin, so that its chance of getting through the stomach to the duodenum, where it normally exerts its function, is very slight, while when combined with pepsin, it must be digested as soon as the mixture becomes warm enough, in or out of the stomach, to carry on the process. Yet we see many preparations of which the chief virtue is supposed to be that they contain *all* the digestive pinciples. These can be active only so far as they contain pepsin, and have no advantage over the simple drug.

It has also been shown that certain substances combined with pepsin in solution render it inert. Alcohol is one, and even in moderation diminishes its action, while, in any quantity, the activity of pepsin is totally prevented. This is a point often lost sight of, and serves as a hint concerning the use of liquors at meals, by dyspeptics.

Pepsin and bismuth are frequently combined when treating dyspepsia, and should always be administered in pill form, owing to the difficulty of keeping both agents together in a permanent solution, one requiring a somewhat acid menstruum, the other a neutral or an alkaline one. Nothing is more common than to see elixirs of pepsin, bismuth and strychnia, which darken, harden, and shrink the albumen placed in them. Liquid preparations are theoretically, if not practically, inconsistent, and will generally be found to be unsatisfactory.

In treating that form of dyspepsia met with in malarious districts and in persons who have passed the autumn months in the country, much good can be obtained from quinine, or a combination of quinine, iron and arsenious acid, given after meals and for a length of time. When using quinine, the pill form is the best that can be resorted to, the soluble-coated pill possessing many advantages over the solution, capsules or wafers. In an article upon quinine, published about four years ago, I called attention to the ready solubility of the bi-sulphate and urged its use, being the most soluble of the quinine salts used in medicine. On this account it is more active and better calculated for administration in pill form than the sulphate ; but it should be borne in mind that as the bi-sulphate of quinine contains a smaller proportion of the pure alkaloid, the amount being only about 60 per cent., the dose should be one-fourth greater than that of the sulphate, in order to represent the same amount of alkaloid.

Iron is a most valuable remedy in the treatment of dyspepsia, but furnishes best results when given toward the termination of a course of treatment.

Active business men, brain-workers, and those who are snbjected to much mental anxiety or overwork, frequently suffer from what is termed " nervous indigestion." To meet this condition we possess a valuable remedy in phosphorus, which should be given in small doses, immediately after meals.

The best form of administering phosphorus is that afforded by the manufactured pill, which possesses many advantages over those made to order in the prescription room. A pill of this kind is exceedingly difficult to make, requiring especial care and skill. The phosphorus should be incorporated with the various ingredients, while in solution, so as to effect a thorough and uniform diffusion ; they should also always be coated, in order to prevent oxidation. I have made some interesting experiments with phosphorus pills manufactured by Schieffelin, of New York, proving that their coating entirely protected the enclosed mass. A pill that had been manufactured a year ago was cut open, and the mass found to be as soft as though made but a few days. Another pill, of the same manufacturer, made eight years ago, was found to be but little harder. Upon placing two of the latter pills in a small quantity of water, the coating was quickly dissolved, the mass broken up, and when placed in a dark room the vial was plainly perceived, owing to the unaltered luminous property of the phosphorus. This simple experiment goes to show that if properly made and coated, a manufactured pill does not become affected by age, nor do the various ingredients become so altered that they lose their medicinal properties. Experience has shown that such pills may be kept for years, even in a very warm climate, and still produce prompt and active effects when administered.

In treating the secondary forms of syphilis, the plan recommended by Dr. Keyes, "of administering mercurials in granules," possesses many advantages over the older method of prescribing the same substance in solution. Intestinal irritation seems to be later and less painful in its manifestations; it is an easier matter to regulate the quantity given when pursuing the "tonic treatment," and the granular form is the most convenient and portable for the patient. Certain cases will be encountered where the symptoms are so pressing that there is not time to get the patient quietly under the influence of the drug, when administered in granules, but fortunately such are not frequent, and can be met by resorting to the older methods until the urgent symptoms abate. The obstinate lesions occurring in the mouth are most successfully treated with granules, the patient being told to allow the little pill to slowly dissolve upon the tongue, thus obtaining the effect of a solution of corrosive sublimate upon the patches, while at the same time the general treatment is pursued.

In a case where the mucous patches in the mouth were very numerous and troublesome, no impression could be made upon them until the plan mentioned was tried, when they disappeared in a short time.

While pursuing the hyoscyamine treatment upon a patient afflicted with mental disease, the manufactured pills were of great advantage, as there was no doubt as to the accuracy of dose, and they were easy to administer.

In a recent conversation with one of the leading alienists of this city, I was informed that he was using pills of this form upon several cases, considering them preferable to the hypodermic method.

When prescribing a medicine so powerful in its influence that doses of from $\frac{1}{250}$ to $\frac{1}{25}$ of a grain are desired, it is almost impossible to obtain the exact amount in each pill, unless a large number are prepared at one time and great care exercised in the manipulation of the mass. With drugs like arsenious acid, aconitia, aloin, atropia, codeia, digitalin, hyoscyamine, morphia, phosphorus, strychnia, and the more powerful mercurials, accuracy of dose and the decreased possibility of a mistake are points in favor of the manufactured pill; while with such remedies as asafœtida, quinia, and others possessing a very unpleasant taste, the coating removes the objection to their use, which patients so frequently make. Little children who struggled and had to be forced to take quinine when prescribed in solution, powders or uncoated pills, will take the drug without objecting, when given in a coated pill.

In this article it has been my intention to point out a few of the advantages possessed by manufactured pills, over those prepared in the old and ordinary manner, and to speak of a few cases in my own practice, where the results from their use have been satisfactory. During the past seven years, the soluble-coated pills made by Schieffelin & Co., of New York, have been the ones prescribed, and I do not hesitate to recommend them to the profession.

The "coating" of pills is a subject of much importance and of especial interest to physicians.

In a future article I shall bring to their notice some facts, to add to those published a number of years ago.—*M. S. French, M. D., in Medical and Surgical Reporter.*

